# Co-staining microplastics with Nile Red and Rose Bengal for improved optical quantification

**DOI:** 10.1038/s41598-025-32829-7

**Published:** 2025-12-23

**Authors:** Benedetta Villa, Gaia Bolla, Isabella Gambino, Elisa Terzaghi, Ginevra Boldrocchi, Enrica Baldini, Filippo Brusa, Stefania Federici, Serena Ducoli, Giovanni Bergna, Alberto Zoccali, Francesca Malpei, Antonio Di Guardo, Roberta Bettinetti

**Affiliations:** 1 Department of Science and High Technology, University of Insubria, Via Valleggio 11, Como, Italy; 2 Department of Human Sciences, Innovation and Territory, University of Insubria, Via Valleggio, 11, Como, Italy; 3 Centro Tessile Serico Sostenibile (CTSS), Via Castelnuovo 3, Como, 22100 Italy; 4 Department of Mechanical and Industrial Engineering, INSTM Unit of Brescia, University of Brescia, Via Branze 38, Brescia, 25123 Italy; 5 Lariana Depur S.p.a, Via Laghetto 1, 22073 Fino Mornasco, Como, Italy; 6 Department of Civil and Environmental Engineering (DICA) - Environmental Section, Politecnico di Milano, Piazza Leonardo da Vinci 32, Milano, 20133 Italy

**Keywords:** Digital microscope, Microplastics, Nile red, Optical quantification, Rose bengal, Visual analysis, Biological techniques, Biotechnology, Chemistry, Environmental sciences, Materials science

## Abstract

Accurate assessment of microplastic (MP) contamination in environmental samples is crucial not only for understanding the scope of this growing environmental threat but also for quantifying its magnitude and enabling proper risk assessment. However, current methodologies for MP quantification often suffer from inaccuracies due to the difficulty in distinguishing plastic particles from natural organic matter, also due to incomplete digestion of natural polymers during sample treatment. Moreover, the techniques commonly employed are highly time-consuming, further limiting their routine application. This research presents an innovative solution for optical microscopy evaluation: a sequential co-staining technique employing Nile Red (NR) and Rose Bengal (RB) to identify natural vs. synthetic polymer fragments as well as false positives. Two experiments were implemented staining natural polymers (cellulose, protein, lignin, and chitin) and synthetic polymers (Polyvinyl Chloride (PVC), Polystyrene (PS), Polyethylene Terephthalate (PET), Polypropylene (PP), Nylon (NY), High-Density Polyethylene (HDPE) and Low-Density Polyethylene (LDPE)) with the two dyes. The results showed that co-staining is an effective way of separating natural and synthetic fragments and a significant improvement in the accuracy of visual MP identification. Additionally, co-staining the same filter allows to obtain relevant time saving as well as reducing counting and identification errors, since no sample exchange is needed. Application of this novel technique will allow for more reliable monitoring of MP concentrations in various environmental matrices, leading to better-informed risk assessments and mitigation strategies.

**Supplementary Information:**

The online version contains supplementary material available at 10.1038/s41598-025-32829-7.

## Introduction

Plastic materials were designed to enhance everyday life; however, the current plastic era has unveiled significant drawbacks^1^. The rapid rise in production and use^2–4^ has led to a corresponding surge in plastic waste^5,6^ and associated negative environmental and health impacts. Despite their crucial role in different modern sectors^7^, plastics are increasingly recognized as a major threat to both the environment and human health due to their extensive use, durability, resistance to degradation, and low rates of recycling^8–10^.

One particularly concerning consequence of plastic proliferation is the emergence of microplastics (MPs). First identified in the literature in the early 2000s^11^, MPs—defined by their size of less than 5 mm^12–14^ and recently of size comprised between 1 mm and 1 μm^15,16^—have garnered increasing attention as they are found in nearly all environmental compartments^1,17,18^.

Although the issue of MPs has gained momentum globally, a lack of standardized methods for analysing these contaminants still exists. Establishing such methods is critical for accurately assessing pollution levels and enabling data comparison^19–21^. MPs analysis is time-consuming and it involves complex morphological (e.g., shape, colour, size) and chemical characterization (such as polymer type and or presence of additives)^22^. Numerous analytical methods are available to assess MPs pollution^23^. However, optical quantification (OQ) is the most accessible and widely explored method because of cost-effectiveness, the ability to provide rapid preliminary screening, and simplicity, making it a practical choice. However, the method disadvantages include loss of small and transparent MPs, low reliability, lack of polymer identification or the weathering state, potential confounding factors due to the presence of natural polymers, often due to incomplete digestion during sample treatment, and operator subjectivity^24,25^.

Therefore, this study aims at improving OQ techniques in terms of efficiency and reliability of data, by introducing a co-staining method involving Nile Red (NR) and Rose Bengal (RB). Staining techniques are a universally shared method introduced to improve simple OQ. Among many dyes, NR is one of the most widely used and most effective dyes to quickly distinguish plastic fragments from those of other origin^26–30^. It is a very accessible fluorescent dye that binds to plastic polymers, allowing for their identification; however, because of its lipophilic character, it can also stain organic materials in some cases, leading to false positives and overestimation of fragment number^29,31^. To mitigate this problem and increase the recovery of MP particles, some researchers suggested other dyes as co-staining method^29,32–34^, although, to our knowledge, co-staining with NR and RB on the same sample was not yet validated nor published. Therefore, in this work, for the first time, we combined the sequential use of NR and RB on the same sample. RB according to the literature is a histological dye, which only stains natural polymers particles, while leaving MPs unstained^29,35^. Moreover, RB has already been used to improve MPs quantification^36–42^. Alonso-Vázquez et al.^35^ used to discriminate natural and synthetic polymers NR and RB on separate filters, with the need of splitting the sample in two subsamples and repeat the measures on both samples. Although this approach could improve the quantification, it may introduce additional variability due to the need of subsampling and repeat the measurements. However, both dyes were never used in sequence on the same filter.

In conclusion, the aim of this work is to furnish the first quantitative results on the co-staining of particles to enhance the rapid and accurate differentiation between plastic particles and natural organic materials, thereby advancing our understanding of MP pollution. The additional advantage of co-staining the same filter is the important time saving obtained and the reduction of errors, since counting is performed on the same set of particles, without sample exchange.

## Materials and methods

### Chemicals and materials

NR-solution was prepared dissolving NR (99% pure, Carlo Erba, Milan, Italy) in acetone (99.95% pure, Carlo Erba, Milan, Italy) at a concentration of 1 g/L. RB (90% pure, Carlo Erba, Milan, Italy) was dissolved in Milli-Q water at a concentration of 200 mg/L^29^. Glass fiber filters (Whatman^®^ glass microfiber grade GF/C filter discs, 1.2 μm pore size, 47 mm diameter) were used to retain and stain MPs. NR staining was performed on MP and natural particles deposited on glass filters using the later selected NR solution and then incubated in an oven for 30 min at 40 °C^31^. Following incubation, filters were rinsed with 10 ml of acetone and 10 ml of Milli-Q to remove excess dye^43^. In case of RB, the solution applied to the filters was allowed to react for 5 min at room temperature^29^, after which filters were rinsed with Milli-Q water to eliminate surplus dye. The stained filters were examined with a digital microscope (Keyence VHX-7000), coupled to a Keyence VHX-7100 camera, Keyence VHX-E100 and VHX-E20 lens, under ultraviolet (UV) light (365 nm). The High-Resolution Medium-Magnification objective lens VHX-E100 (100× to 500×) offers a resolution of 2 μm, enabling the observation of MPs ranging from 5 mm to 2 μm^44^.

Synthetic polymers utilised: Polyvinyl Chloride (PVC), Polystyrene (PS), Polyethylene Terephthalate (PET), Polypropylene (PP), Nylon (NY), High-Density Polyethylene (HDPE) and Low-Density Polyethylene (LDPE). All these polymers were transparent, except for PVC fragments, which were black/grey (see in supplementary materials Table SI-1.a). MPs of different polymers were obtained by cutting plastics in small pieces from everyday products. Natural polymers were obtained from different originating materials: cotton thread (source of cellulose fibers, from now on “cellulose 1”), toilet paper (additional source of cellulose fibers, from now on “cellulose 2”), wool thread (source of protein based fibers, from now on “protein”), sawdust (source of lignin, from now on “lignin”), and fungal mycelium (source of chitin^45^, from now on “chitin”) (Table SI-1.b). Cotton and wool threads were purchased at knitting stores, sawdust was obtained from locust wood, commercial edible mushrooms (*Boletus edulis*) and white (unstained) toilet paper were obtained at local stores. All particles created and analysed had dimensions lower than 5 mm, trying to cover as much as possible the entire range: 1 μm – 5 mm (Table SI-1).

### Experimental design of OQ staining comparison

**Fig. 1:**
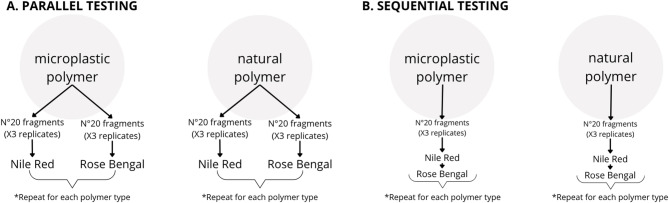
Procedure followed to validate the method of co-staining with NR and RB.

Initially, four NR concentrations were tested (0.001, 0.01, 0.1, and 1 g/L) to find the concentration at which an optimal fluorescence was obtained. Later, polymers (natural and synthetic) were added with tweezers to the glass fiber filters and stained with NR or/and RB. Nine replicates of 20 fragments each were used for staining each polymer. Staining was performed separately or sequentially (Fig. 1) in two different experiments to evaluate how it would affect different materials. The parallel test was carried out by staining each polymer sample first with NR and then, later, with RB. This means that each polymer was stained with only one dye at a time, and the two staining procedures were performed independently of each other (Fig. 1A). Sequential staining was instead performed each polymer initially with NR and right after with RB (Fig. 1B).

### Selection of OQ conditions

**Fig. 2:**
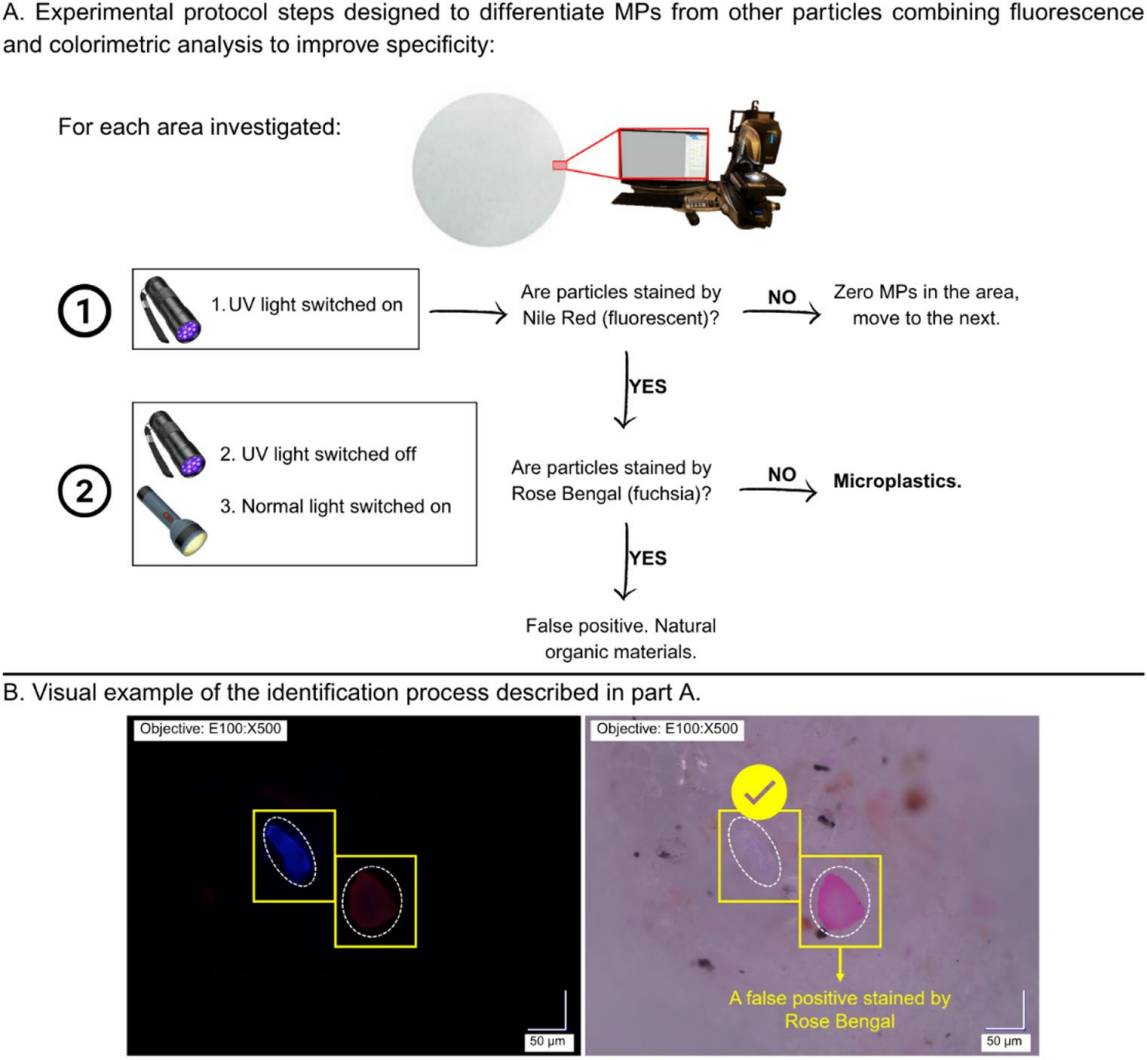
(**A**) Step-by-step method for MP optical quantification; (**B**) Example showing the detection of a MP fragment and a false-positive particle following visual inspection under UV and normal light.

In the counting procedure, the filter area visible on the monitor (Fig. 2A) was first investigated under the UV light, allowing for the detection of NR stained fluorescent particles, potentially being considered MPs. Subsequently, the UV light was switched off to observe the same particles under visible light. When a particle, previously counted as MP under UV light, appeared fuchsia coloured, it was discarded, since it could indicate a natural origin (Fig. 2B).

### Preparation of environmental samples for OQ validation

Four wastewater samples were collected on multiple times in 2024 at two different Wastewater Treatment Plants (WWTP). These samples were processed to obtain comparable subsamples as part of a larger intercalibration exercise coordinated by colleagues at the University of Brescia, within the framework of the European Union LIFE Cascade project (https://lifecascade.eu). The four water samples were obtained from different stages of two wastewater treatment plant (Table S1–S5) and were analysed with several techniques, including FTIR and OQ.

The original water samples were processed to reduce organic matter and separate MPs. More specifically, samples underwent an extraction and digestion process, performed according to different methodologies^46–49^. In short, samples processing involved multiple steps, from oxidative treatments (Fenton’s oxidation) to remove organic matter, density separation with ZnCl₂, and sample washing^50^. For the intercalibration aliquots of 50 mL of water containing MPs were delivered to each laboratory.

Quantification via microFTIR (Perkin Elmer Spotlight 400 System, paired to silver membrane filters, Sterlitech, 13 mm diameter, 5 μm pore size) was carried out by manually inspecting the entire filter area through point-by-point spectral acquisition, generally in a scan range 4000 –650 cm^− 1^ with 8 or 16 scans per particle, in reflectance mode. In this context, the primary aim was not the detailed chemical identification of each particle, but rather the estimation of the total number of MPs presents in the sample. Depending on the distribution and concentration of the MPs, the nature of the particles and/or fibers larger than 20 μm or of the whole filter or a portion of it is identified. The FTIR spectrum is interpreted and compared with reference spectra present in the libraries (Polymers Library and laboratory database) in order to identify its nature: the spectral quality and matching score (> 60%) with the reference library was taken into account, but the interpretation must also take into account the possible environmental alterations and degradations of the polymers that the MPs are made of. Particles were counted only when their infrared spectra showed clear features compatible with synthetic polymers. Although this manual approach is time-consuming and limited to particles above a certain size threshold (typically around 20–30 μm), it allows for a consistent and reasonably accurate quantification of MPs.

### Quality assurance and quality control

A number of procedures were strictly followed to avoid contaminations (filtered solutions, no plastic items, clean glassware, clean gloves, clean laboratory surfaces, only cotton clothes). During the analysis, laboratory blanks were prepared following the same procedure used for filters containing polymers. Additionally, air deposition blanks were conducted by leaving a filter (similar to those employed in the MP staining) exposed to air throughout the entire analysis process, so to control the contamination due to cotton lab coats and clothes. Only Milli-Q water was utilized as rinsing solution, and all reagents were filtered on 1.2 μm glass fiber filters prior to use to eliminate particles and minimize the risk of contamination. During FTIR analysis, for each batch of samples, a blank consisting of 50 ml of Milli-Q water was analyzed in the same way as the samples, with the same reagents, materials and the same apparatus. The silver membrane filter was analyzed, and the particles identified.

Blanks showed a mean value of 5.6 ± 2.6 MPs, generally about 3% of the fragments detected in the sample. Each blank value was subtracted from the corresponding sample result. Therefore, data reported in the results and discussion section are to be considered blank subtracted.

### Statistical analysis

T-test and One-way ANOVA were performed to test differences among averages, using XLSTAT 2025.1.3 software (Addisoft, USA) in Microsoft Excel.

## Results and discussion

Although polymers dyeing could be influenced by a number of factors derived by the nature of polymer surface^21,22^ (surface polarity, hydrophobicity, crystallinity, etc.), it was reported that MPs in water may act as a colloidal system^51^. In this case the surface interaction could be influenced by surface properties, particle size, water composition, ionic content, and temperature^51^ as well as surface roughness and other surface characteristics, as recently outlined by Burrows and coworkers^52^ who call for additional studies on this subject. However, we decided to concentrate our efforts on the selection of the best conditions for MP detection with the combination of the two staining techniques, as a first step.

### Evaluation of best OQ conditions

The use of a digital microscope equipped with high-resolution objective lens allowed the observation of MPs within the 2–20 μm range, in fact MPs as small as 2 μm were identified. This is particularly challenging for OQ and MPs in this last size range are usually lost^26^. Staining intensity was quantified by measuring the contrast ratio between the luminance of the polymer colour and the luminance of the background colour^53,54^. Each colour was expressed using a hexadecimal code (HEX) obtained from a single pixel. The resulting colour in HEX code was a mean between forty measurements, which were chosen avoiding fragment edges and selecting areas where colour appeared homogeneous. To indicate if a fragment was stained or not, a value of 2.5:1 was identified as a visibility threshold. This threshold was selected as an empirical, operational value, based on the comparison of the concentrations needed to detect polymer fluorescence, while not impairing its recognition due to excess dye. A ratio above this value indicates clear contrast and a colour that is easily visible, therefore polymers were considered coloured. Colouration is considered absent when the value was below the threshold of 1.1:1^53^, indicating that > 1.1:1 and < 2.5:1 was identified as range where the colouration was present but slightly visible.

### Optimization of NR concentration

**Fig. 3:**
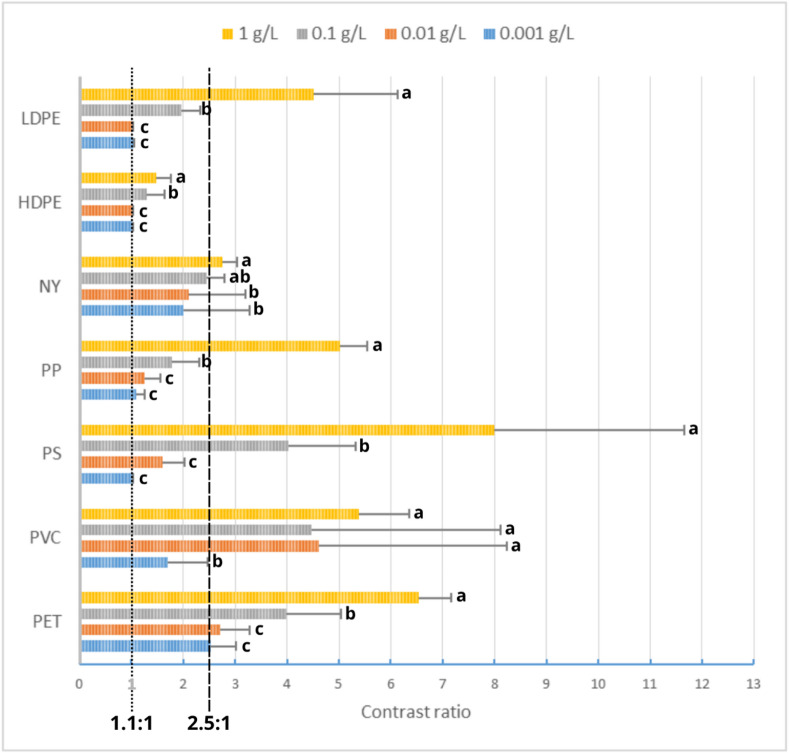
Staining effectiveness calculated with a contrast ratio between background and the polymer colour after NR staining with four different concentrations (0.001 g/L; 0.01 g/L; 0.1 g/L; 1 g/L). Different colours represent different tested concentrations; different small letters represent statistically different contrast ratio per polymer type (*p* < 0.05). For individual p-values see Supplementary Information Text 2.

Before starting the method validation, different concentrations of NR solution were tested to determine the optimal concentration that provides a strong fluorescence signal for all MPs polymers observed under UV light (Fig. 3). In the scientific literature, various concentrations of NR are reported for the quantification of MPs. Shruti et al.^31^ described a range of NR concentrations between 0.0005 g/L and 10 g/L. They also mentioned that the most used concentration was 1 g/L, as it was reported in many studies. Maes et al.^27^ suggested that the concentrations range should be comprised between 0.001 g/L and 1 g/L to have a good fluorescence intensity. Therefore, the concentrations tested were: 0.001 g/L, 0.01 g/L, 0.1 g/L, 1 g/L. The concentration of 0.001 g/L was tested even if Prata et al.^43^ already proved that NR solution cannot be diluted under 0.01 g/L to obtain a sufficient staining.

The results of NR staining showed significant differences among staining intensities per polymer class: as presented in Fig. 3, staining intensities at a concentration of 1 g/L are generally statistically significantly different (group a, with the exception of NY) and result in a distinctly visible and enhanced colouration compared to the other tested concentrations.

The NR concentration of 0.001 g/L did not colour most of the polymers, as shown by Fig. 3 and the contrast ratio values in Table SI-4. This NR concentration did not stain PS, PP, HDPE and LDPE, which were below the 1.1:1 contrast ratio threshold, a threshold that indicates no visible contrast between the colour and its background (Fig. 3). NY and PVC were lightly coloured (2.0:1 and 1.69:1, respectively). PET was the only polymer being coloured, its contrast ratio of 2.54:1 was above the level of 2.5:1. This agrees with Prata et al. ^43^ that already suggested that the NR concentration should not be lower than 0.01 g/L to be efficient in staining.

Our results also proved that the level of 0.01 g/L was not optimal for obtaining a good fluorescence, in fact, staining only improved in PS and PP fragments, which have changed from uncoloured to slightly coloured (Fig. 3 and Table SI-4), and PVC, whose colour has improved until it became clearly visible (contrast ratio of 4.61:1).

The increase in concentration, up to 0.1 g/L, resulted in improved colouration and thus fluorescence (Fig. 3 and Table SI-4). PS fragments became visible, and HDPE and LDPE fragments displayed minimal pigmentation. While PET, PVC and PS presented a strong colouring.

Finally, best results were obtained with the concentration of 1 g/L, as shown in Fig. 3 and Table SI-4. This concentration was chosen for the final staining tests. PVC, PS, PET, PP, NY and LDPE showed a good fluorescence. Only HDPE fragments demonstrated a reduced colouration (1.47:1), although all fragments were stained, despite their varying sizes.

### Intrinsic fluorescence

Polymers, either natural or synthetic, may possess an intrinsic fluorescence (IF), appearing under UV light even without any staining. Natural materials may exhibit IF which could add up to the NR induced fluorescence, as mentioned previously^29^. Table 1 shows the IF of natural and synthetic polymers, classified according the three levels of contrast ratio mentioned before (< 1.1:1; between 1.1: 1 and 2.5:1 and > 2.5:1) while Table SI-1 shows the visual results and Table SI-2 and SI-3 the numerical values.

**Table 1:**
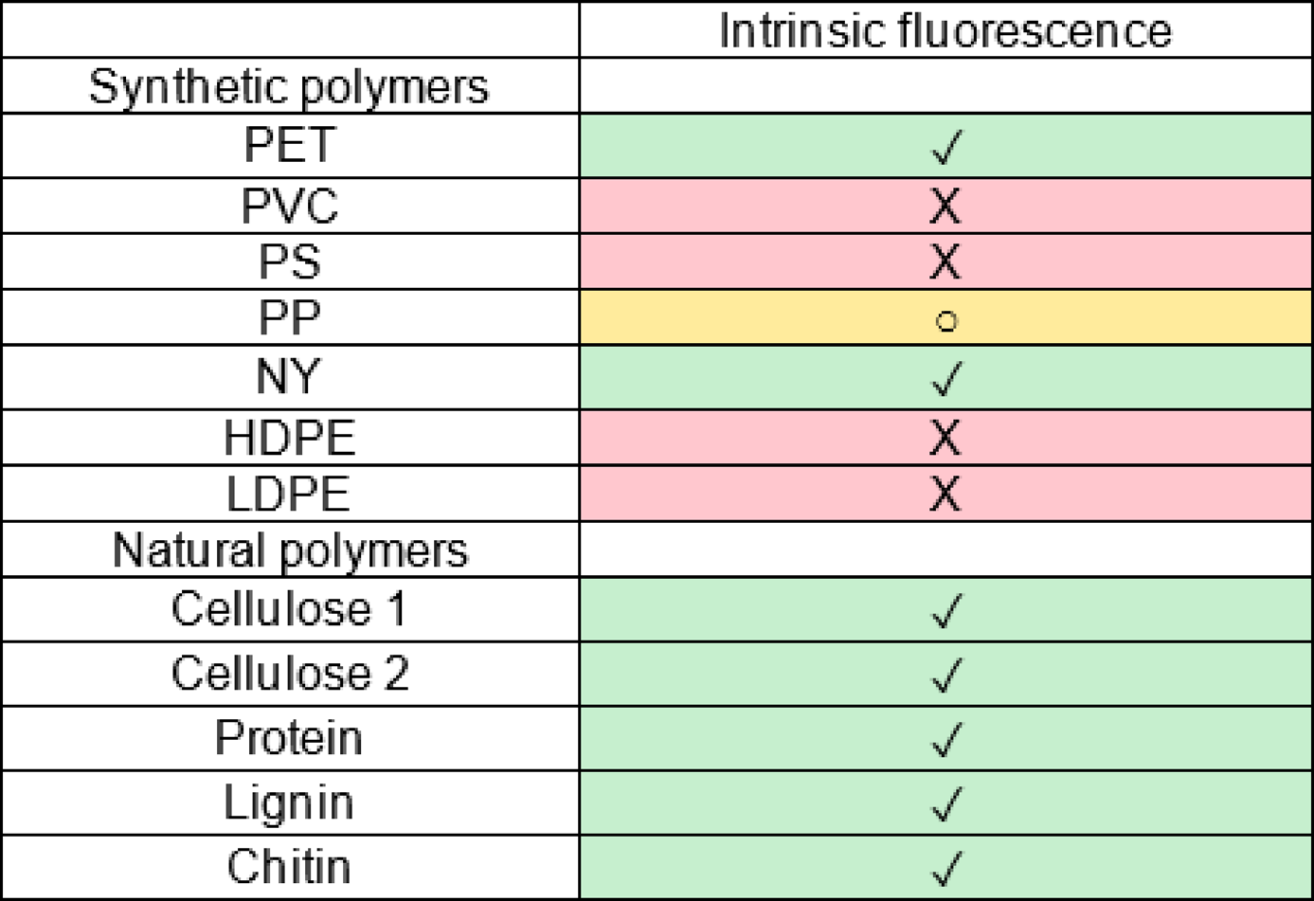
Intrinsic fluorescence of synthetic and natural polymers.

IF is represented by three symbols and colours: ✓ (on green background) indicates the presence of IF (contrast ratio >2.5:1), ○ (on yellow background) indicates that polymers had a low level of IF (contrast ratio between 1.1:1 and 2.5:1), and X (on red background) indicates that polymers did not present appreciable IF (contrast ratio 1.1:1).

The results show that IF was evident for all natural polymers, with all contrast ratios above the threshold level of 2.5:1. However, also PET and NY showed high IF (above 2.5:1) while PP to a lesser extent (Table 1 and Table SI-3).

### Separate MP staining

Separate staining (Fig. 1A) was tested for each polymer in the first experiment when all polymers were separately stained with NR and RB (Table 2) on different filters. Polymers were considered visible and countable when the contrast ratio thresholds > 2.5:1, with the exception of HDPE, for which lower contrasts ratios were observed (between 1.1:1 and 2.5:1).

**Table 2:** Percent staining of fragment with separate use of NR and RB (average of 60 fragments).

		Nile red	Rose Bengal
		Percent stained ± CV	Percent stained ± CV
Synthetic polymers			
PET		100	0
PVC		87 ± 10	0
PS		100	0
PP		100	0
NY		100	0
HDPE*		100	0
LDPE		90 ± 5	0
Natural polymers			
Cellulose 1		90 ± 10	100
Cellulose 2		100	100
Protein		47 ± 13	100
Lignin		85 ± 5	100
Chitin		95 ± 5	100

CV is coefficient of variation. *Although HDPE contrast ratio was lower, fragments were still countable.

The synthetic polymers were generally dyed with NR, as shown before (Fig. 3), with PET, PS, PP, NY, and HDPE fragments all (100%) stained. The efficacy of NR was slightly smaller with PVC and LDPE fragments but well above 85%. However, RB did not colour synthetic polymers, as expected.

Natural polymers instead were all 100% coloured by RB, while some exhibited fluorescence with NR, especially for cellulose 1 and 2 (more than 90% above of 2.5:1 contrast ratio threshold). Lignin and chitin follow with more than 85%, while protein fibers were coloured at about 50%. These results may be due to the intrinsic fluorescence shown in Table 1 but also to some increased fluorescence (especially for cellulose 2, lignin, and chitin) caused by NR (Table SI-2). The NR treatment sometimes caused a shift in colour, especially for lignin (from green to yellow and red) and chitin (from blue to dark violet).

### Sequential MP staining

The second experiment consisted in testing NR and RB sequentially on the same filter (Fig. 1B). Results are presented in Table 3, classifying the results with the contrast ratios threshold of the previous experiment. The NR counts are consistent with those in Table 2: this dye proved to be highly effective in MPs-staining, particularly for PET, PS, PP, NY, and HDPE particles with an efficiency of 100%. PVC and LDPE fragments demonstrated slightly lower staining efficiency, with about 90% of fragments stained. As seen in Table 3, natural materials exhibited high levels of NR staining, with average dyeing rates of: cellulose (1 and 2) of about 100%, lignin and chitin more than 80%, while protein again about 50%. These high levels of fluorescence in natural fragments could again be generally attributed to NR colouration (except for protein and chitin) and the intrinsic fluorescence of materials (Table 1 and Table SI-2).

After NR staining, all replicates were subsequently dyed with RB. Despite the sequential application of both dyes, no interference could be observed in RB staining. Furthermore, RB was confirmed as being highly effective in staining natural components (100% colouring efficacy), leaving MPs unstained (Table 3).

**Table 3:** Percentage staining of fragments with sequential use of NR and RB (average of 60 fragments).

		Nile red	Rose Bengal
		Percent stained ± CV	Percent stained ± CV
Synthetic polymers			
PET		100	0
PVC		88 ± 10	0
PS		100	0
PP		100	0
NY		100	0
HDPE*		100	0
LDPE		91 ± 3	0
Natural polymers			
Cellulose 1		98 ± 3	100
Cellulose 2		100	100
Protein		47 ± 8	100
Lignin		88 ± 3	100
Chitin		82 ± 8	100

CV is coefficient of variation. *Although HDPE contrast ratio was lower, fragments were still countable.

The results of the two staining experiments showed no significant differences. Therefore, even though fluorescence could be evident for some natural polymers, due to NR staining or intrinsic fluorescence (potentially interpreted as MPs), RB proved to be an effective method for distinguishing the presence or absence of plastic components. Therefore, the sequential staining technique can be described as an improved and effective way of discriminating natural and synthetic polymer fragments.

### Validation with environmental samples

**Fig. 4:**
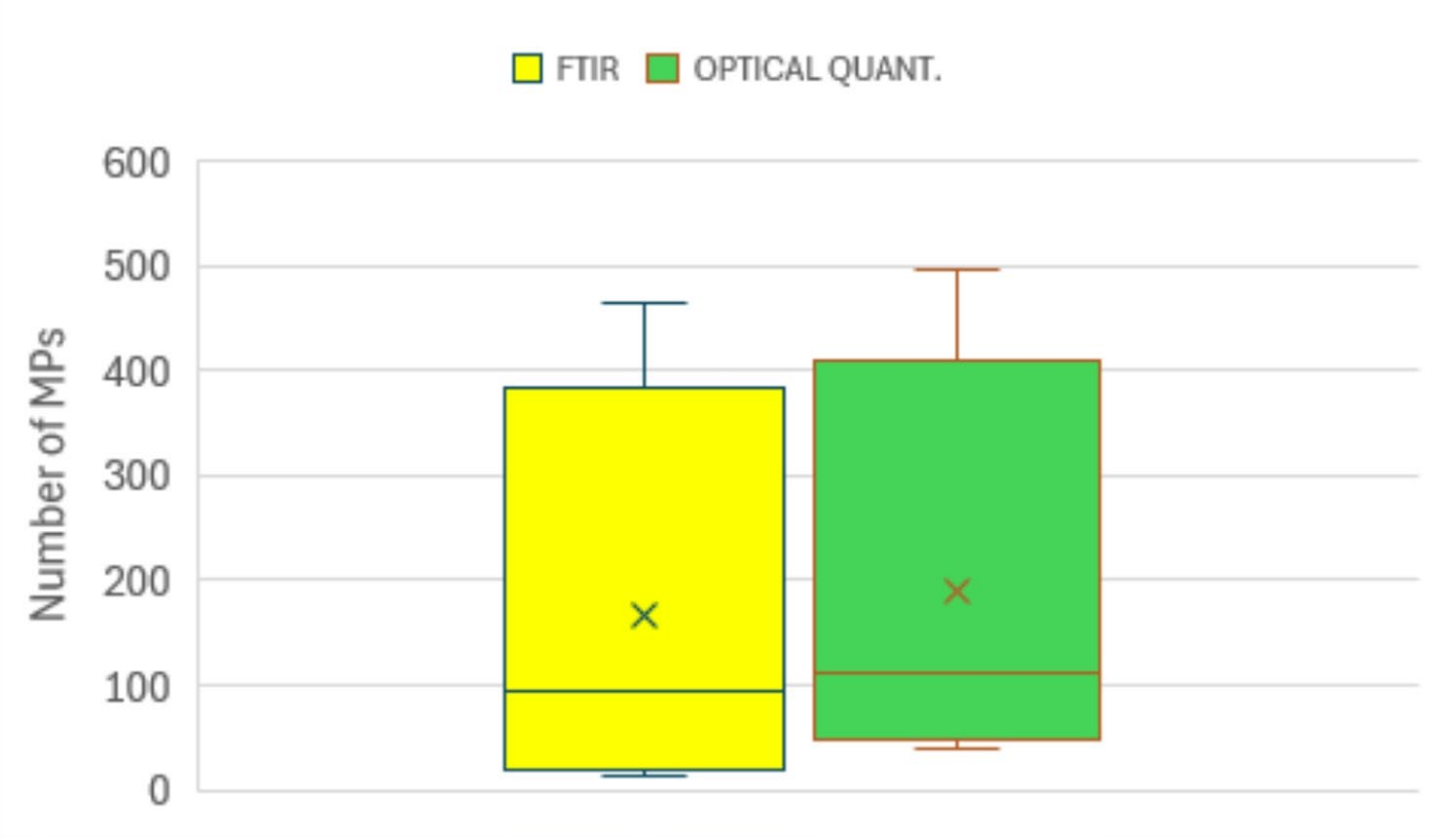
Box plots of microplastic counts measured by FTIR and OQ across four wastewater treatment samples. “x” indicates the averages, the horizontal straight line within the box indicates the median, while lower and higher whiskers indicate, respectively, the 10° and 90° percentile.

The new combined staining technique presented here was further evaluated with environmental water samples from a wastewater treatment plant, which pose additional challenges due to their complex matrices, the presence of organic debris, and the broader variability in particle morphology and composition. These factors often hinder both the detection and the accurate discrimination between synthetic and natural particles, thus making robust and selective staining protocols particularly valuable. Figure 4 shows the results of the comparison between the MP counts obtained from four wastewater samples using both microFTIR analysis and OQ with co-staining (Table SI-5), as part of the intercalibration exercise. The average number of MPs detected per sample was 166 ± 206 with FTIR and 189.5 ± 210 with OQ. A paired-t indicated no statistically significant difference between the averages of the two techniques (p-value > 0.05), suggesting that the new staining-based method yields comparable results in terms of MP counts. This observation is further supported by the boxplot in Fig. 4, which shows overlapping distributions, including similar averages, medians, and interquartile ranges. These results corroborate the reliability of OQ in comparison with one of the most common detection techniques (FTIR).

It is important to note that the comparison here is limited to the numerical quantification of suspected MP particles and does not provide any direct information on their chemical identity or weathering state. The optical method relies solely on morphological features and differential staining response; thus, false positives or negatives cannot be entirely ruled out. Nevertheless, the observed consistency with FTIR-based counts fully supports the co-staining approach as a rapid screening tool for MP enumeration in environmental samples.

## Conclusions

This study demonstrates the successful application of a combined NR and RB co-staining method to improve the OQ of MPs.

The effectiveness of the method has also been tested on real environmental matrices. For optimal results with this method, a thorough digestion is crucial, e.g., using hydrogen peroxide, which has been proven to be an excellent method to digest natural organic matter without affecting MPs^55,56^. Incomplete digestion or an excess of organic material on the filter can result in RB staining, leading to high background noise and making the distinction between plastic and natural particles difficult. Particularly relevant is the impact of aged MPs, which due to surface alterations and biofilm formation over time, can more easily attract the RB dye, complicating the interpretation of the results. Therefore, careful sample selection and efficient digestion protocols are essential.

It is important to emphasize that the method provides a reliable way for the rapid differentiation between plastic and natural particles, but it does not distinguish among the polymer types present in the sample. For this purpose, additional qualitative or quantitative techniques are required, such as FTIR, Raman spectroscopy or PY-GC-MS (pyrolysis-gas chromatography-mass spectrometry).

However, this technique paves the way for more effective assessments of MPs, which can be also done automating the acquisition process with open-source software tools such as ImageJ^28,43,57^. For example, Prata et al.^28,43^ developed the Microplastics Visual Analysis Tool (MP-VAT), an ImageJ plugin designed for automatic characterization and quantification of MPs. Incorporating machine learning and artificial intelligence approaches into these workflows holds great promise to further enhance detection accuracy, enable real-time analysis, and facilitate large-scale of MP environmental monitoring.

## Supplementary Information

Below is the link to the electronic supplementary material.

Supplementary Material 1

## Data Availability

All relevant data are included in the paper and in the supporting information.
