# Supplementary material for: Co-staining microplastics with Nile Red and Rose Bengal for improved optical quantification
Source: Sci Rep. 2025 Dec 23;16:2917. doi: 10.1038/s41598-025-32829-7 (PMC12830938; doi:10.1038/s41598-025-32829-7)
Supplement: Supplementary file 1 — Supplementary Material 1 [file 41598_2025_32829_MOESM1_ESM.docx]

**Supplementary information for**

**Co-staining microplastics with Nile Red and Rose Bengal for enhanced optical microscopy quantification: method development and validation**

Benedetta Villa^a,b^, Gaia Bolla^a^, Isabella Gambino^a^, Elisa Terzaghi^a^, Ginevra Boldrocchi^b^, Enrica Baldini^c^, Filippo Brusa^c^, Stefania Federici^d^, Serena Ducoli^d^, Giovanni Bergna^e^, Alberto Zoccali^f^, Francesca Malpei^f^, Antonio Di Guardo^a^ and Roberta Bettinetti^b^*

^a^ Department of Science and High Technology, University of Insubria, Via Valleggio 11, Como, Italy

^b^ Department of Human Sciences, Innovation and Territory, University of Insubria, Via Valleggio, 11, Como, Italy

^c^Centro Tessile Serico Sostenibile (CTSS), Via Castelnuovo 3, 22100, Como, Italy

^d^Department of Mechanical and Industrial Engineering, INSTM Unit of Brescia, University of Brescia, Via Branze 38, 25123 Brescia, Italy

^e^Lariana Depur S.p.a., Via Laghetto 1, 22073 Fino Mornasco, Como, Italy

^f^Department of Civil and Environmental Engineering (DICA) - Environmental Section, Politecnico di Milano, Piazza Leonardo da Vinci 32, 20133 Milano, Italy

*Corresponding author: roberta.bettinetti@uninsubria.it

Table of Contents

[Table SI-1.a. 2](#_Toc215569219)

[Table SI-1.b. 3](#_Toc215569220)

[Table SI-2. 4](#_Toc215569221)

[Table SI-3. 4](#_Toc215569222)

[Table SI-4. 4](#_Toc215569223)

[Table SI-5. 5](#_Toc215569224)

[Text 1. 6](#_Toc215569225)

[Text 2. 6](#_Toc215569226)

Table SI-1.a. Synthetic polymers observed with digital microscope under normal light, UV light, without staining and after dying process with NR (1 g/L) and RB.

| **Polymers** | **Size range (µm)** | **No staining** | | **Staining** | |
| --- | --- | --- | --- | --- | --- |
|  |  | **Normal Light** | **UV light (Intrinsic Fluorescence)** | **Nile Red**  **(UV light)** | **Rose Bengal**  **(Normal light)** |
| PET | 138 - 3988 | 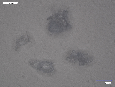 | 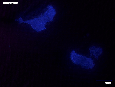 | 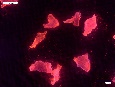 | 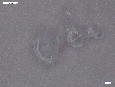 |
| PVC | 2 – 3251 | 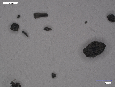 | X | 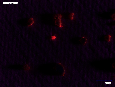 | 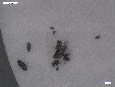 |
| PS | 185 - 4731 | 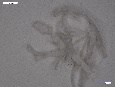 | X | 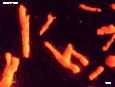 | 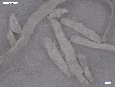 |
| PP | 126 - 3881 | 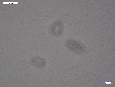 | 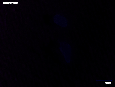 | 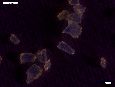 | 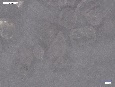 |
| NY | 354 - 4518 | 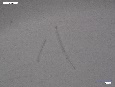 | 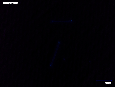 | 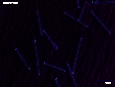 | 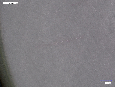 |
| HDPE | 68 - 3989 | 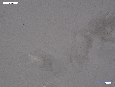 | X | 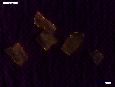 | 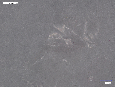 |
| LDPE | 388 - 4070 | 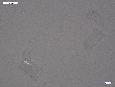 | X | 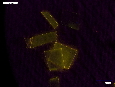 | 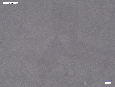 |

Note: yellow and blue bars in the right bottom corner indicate, respectively, scales of 4 and 2 mm

Table SI-1.b. Natural polymers observed with digital microscope under normal light, UV light, without staining and after dying process with NR (1 g/L) and RB.

| **Polymers** | **Size range (µm)** | **No staining** | | **Staining** | |
| --- | --- | --- | --- | --- | --- |
|  |  | **Normal Light** | **UV light (Intrinsic Fluorescence)** | **Nile Red**  **(UV light)** | **Rose Bengal**  **(Normal light)** |
| “Cellulose 1” | 136 – 2572 | 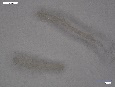 | 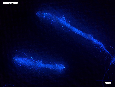 | 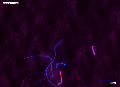 | 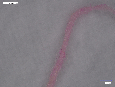 |
| “Cellulose 2” | 137 – 2648 | 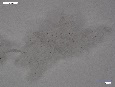 | 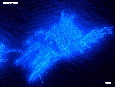 | 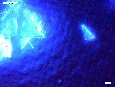 | 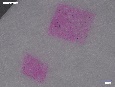 |
| “Protein” | 209 – 3532 | 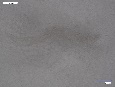 | 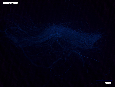 | 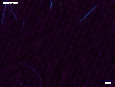 | 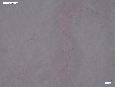 |
| “Lignin” | 16 – 1479 | 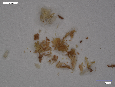 | 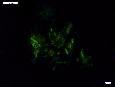 | 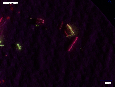 | 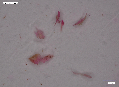 |
| “Chitin” | 2 - 3158 | 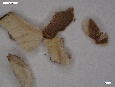 | 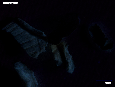 | 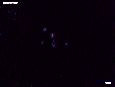 | 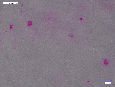 |

Note: yellow and blue bars in the right bottom corner indicate, respectively, scales of 4 and 2 mm

Table SI-2. Natural polymers intrinsic fluorescence, post-NR (1 g/L) and RB colour staining, and corresponding quantification (contrast ratio) (colours are expressed in hexadecimal (HEX) code).

| **Polymers** | **Intrinsic Fluorescence Colour** | **Contrast Ratio (Intrinsic Fluorescence)** | **Colour after NR-staining** | **Contrast Ratio (after NR)** | **Colour after RB-staining** | **Contrast Ratio (after RB)** |
| --- | --- | --- | --- | --- | --- | --- |
|  |  |  |  |  |  |  |
| Cellulose 1 | Blue #1c5df5 | 3.81:1 | Blue #70fff4 | 3.57:1 | Fuchsia #874A6F | 2.53:1 |
| Cellulose 2 | Blue #0bb8fc | 7.85:1 | Blue #88fbf6 | 14.54:1 | Fuchsia #723C6A | 2.64:1 |
| Protein | Blue #064BB1 | 2.52:1 | Blue #2E49B8 | 2.63:1 | Fuchsia #735064 | 2.6:1 |
| Lignin | Green #30761d | 3.7:1 | Yellow #efdf7f | 15.42:1 | Fuchsia #7b2f4b | 2.92:1 |
|  |  |  | Red #a61b56 | 2.89:1 |  |  |
| Chitin | Blue #365972 | 2.76:1 | Dark violet #9878a6 | 5.44:1 | Fuchsia #6C3364 | 2.73:1 |

*Notes:* >2.5:1 coloured (green).

Table SI-3. Intrinsic fluorescence of synthetic polymers and corresponding quantification (contrast ratio).

| **Polymers** | **Intrinsic Fluorescence** | **Contrast Ratio** |
| --- | --- | --- |
|  |  |  |
| PET | Blue #2235E2 | 2.62:1 |
| PVC | #010103 | 1:1 |
| PS | #010004 | 1.02:1 |
| PP | Blue #0E0655 | 1.15:1 |
| NY | Blue #0044C2 | 2.54:1 |
| HDPE | #020003 | 1.02:1 |
| LDPE | #010005 | 1:1 |

*Notes:* >2.5:1 coloured (green); 1.1:1 <X< 2.5:1 slightly coloured (orange); and <1.1:1 not coloured (red).

Table SI-4. Contrast ratio of synthetic polymers (and background) after NR-staining.

| **Polymers** | **Contrast Ratio after**  **NR-staining (0.001 g/L)** | **Contrast Ratio after**  **NR-staining (0.01 g/L)** | **Contrast Ratio after**  **NR-staining (0.1 g/L)** | **Contrast Ratio after**  **NR-staining (1 g/L)** |
| --- | --- | --- | --- | --- |
| PET | 2.54:1 | 2.71:1 | 4.00:1 | 6.53:1 |
| PVC | 1.69:1 | 4.61:1 | 4.47:1 | 5.39:1 |
| PS | 1.02:1 | 1.59:1 | 4.03:1 | 8.00:1 |
| PP | 1.09:1 | 1.25:1 | 1.78:1 | 5.02:1 |
| NY | 2.00:1 | 2.10:1 | 2.45:1 | 2.75:1 |
| HDPE | 1.02:1 | 1.02:1 | 1.30:1 | 1.47:1 |
| LDPE | 1.03:1 | 1.02:1 | 1.97:1 | 4.52:1 |

*Notes:* >2.5:1 coloured (green); 1.1:1 <X< 2.5:1 slightly coloured (orange); and <1.1:1 not coloured (red).

Table SI-5. Information on the four environmental samples from two different Wastewater Treatment Plants.

| **Sample** | **WWTP** | **Treatment phase** | **FTIR (Number of fragments)** | **Optical Quantification (Number of fragments)** |
| --- | --- | --- | --- | --- |
| 1 | 1 | Biological treatment out | 144 | 151 |
| 2 | 1 | Chemical treatment out | 12 | 72 |
| 3 | 2 | Pre-treatment out | 464 | 496 |
| 4 | 2 | Biological treatment out | 44 | 39 |

Text 1. Contrast Ratios (referring to **Table S2**)

The contrast ratios are used to quantify staining levels and polymers intrinsic fluorescence.

The three contrast ratios are as follows:

1. **Contrast ratio between intrinsic fluorescence and background**: This ratio quantifies the intensity of the polymer's natural fluorescence in comparison to the background. A higher contrast ratio indicates stronger intrinsic fluorescence relative to the background.
2. **Contrast ratio between colour after NR-staining and background**: This ratio quantifies the intensity of the staining caused by NR in relation to the background. A higher contrast ratio indicates stronger NR-staining.
3. **Contrast ratio between colour after RB-staining and background**: This ratio quantifies the intensity of the staining caused by RB in relation to the background. A higher contrast ratio indicates stronger RB-staining.

These contrast ratios help evaluate the effectiveness of staining and also the level of polymers intrinsic fluorescence.

Text 2. P-values (referring to **Figure 3**)

- **PET**: There is no significant difference between 0.01 and 0.001 (p = 0.701), which is why they share group c. All other pairwise comparisons among groups a, b, and c are highly significant (p < 0.0001), confirming three statistically distinct groups for PET concentrations in this dataset.
- **PVC**: From the Tukey (HSD) output for PVC, there are two significantly different groups: A (1, 0.01, 0.1 g/L) and B (0.001 g/L). The corresponding p-values for the pairwise comparisons are:
  - 1 vs 0.001: p < 0.0001 (significant; A vs B)
  - 0.01 vs 0.001: p < 0.0001 (significant; A vs B)
  - 0.1 vs 0.001: p < 0.0001 (significant; A vs B)

All other pairwise comparisons are not significant:

- - 1 vs 0.1: p = 0.369 (both in group A)
  - 1 vs 0.01: p = 0.599 (both in group A)
  - 0.01 vs 0.1: p = 0.994 (both in group A)
- **PS:** There is no significant difference between 0.01 and 0.001 (p = 0.562), which is why they share group c. All other pairwise comparisons among groups a, b, and c are highly significant (p < 0.0001).
- **PP:** There is no significant difference between 0.01 and 0.001 (p = 0.349), which is why they share group c. All other pairwise comparisons among groups a, b, and c are highly significant (p < 0.0001).
- **NY:** There is no significant difference between 1 and 0.1 (p = 0.414) and a significant difference between 1 to 0.01 (p = 0.006), and 0.001 (p = 0.001). Moreover, 0.1 is not statistically different from 0.01 (p = 0.280) and 0.001 (p = 0.101). Finally, there is not a difference between 0.01 and 0.001 (p = 956). There are two different groups a (1, 0.1) and b (0.1, 0.01, 0.001).
- **HDPE:** There is no significant difference between 0.01 and 0.001 (p = 1), which is why they share group c. All other pairwise comparisons among groups a, b, and c are highly significant (p < 0.0001; and p = 0.003 for 1 vs 0.1).
- **LDPE:** There is no significant difference between 0.01 and 0.001 (p = 1), which is why they share group c. All other pairwise comparisons among groups a, b, and c are highly significant (p < 0.0001).
